# Comment on “Association Between Injury Etiology and Scar Outcomes: A Retrospective Clinical Study”

**DOI:** 10.1111/jocd.71170

**Published:** 2026-08-30

**Authors:** Meerab Akhtar Abbasi, Kumar Singh, Maheen Hamid

**Affiliations:** ^1^ Dow Medical College (DMC) Karachi Pakistan; ^2^ Multan Medical and Dental College Multan Pakistan

Dear Editor,

1

We read the article ‘Association between Injury Etiology and Scar Outcomes’, published by Huang et al., in Journal of Cosmetic Dermatology, with great interest. Scar is fibrous connective tissue which replaces non‐injured tissue following injury [1]. We commend the authors for this valuable study, which underscores the importance of injury etiology as an important determinant of scar outcomes and explores the role of injury etiology in early clinical risk stratification [2]. Some methodological issues that could increase the generalizability of the results, however, remain unaddressed.

Although the authors considered many important variables like age, gender, scar stage, and injury etiology, we believe that some other patient‐specific variables such as nutritional status and certain systemic factors that were not taken into account could have affected scar outcomes.

Recent literature also indicates that poor nutrition can cause healing to be impaired, as it can affect the functioning of cells (fibroblasts) that have a central role in the healing process and in the formation of scars [3]. Furthermore, there is evidence from previous studies suggesting that some systemic factors such as the circulating cytokines, endocrine factors such as vitamin D status and the renin‐angiotensin system, and lifestyle factors such as smoking, diet, psychological stress and exercise may be involved in scar formation and its progression [4]. Thus, the exclusion of these factors may have led to confounding and therefore impacted the reported association between etiology and scar outcomes.

Moreover, although the post‐injury intervention and the effect it had on scar outcome were evaluated in a descriptive manner within this cohort, these paths toward interventions were not included in the final architecture of the prognostic nomogram. Based on management algorithms for hypertrophic scars and keloids, the initial management should be based on the individual patient's risk profile and, ideally, adjusted to the dynamic situation to prevent abnormal fibroblastic proliferation. For instance, silicone gel sheeting treatment, pressure garments, or specific intralesional treatment of high‐risk wounds can yield far greater preventative results when it is treated early, before the formation of abnormal scar tissue [5]. The predictive model is based solely on baseline characteristics and does not take into account the fact that some patients had early preventive care and others had late therapeutic care; therefore, its clinical predictive accuracy is called into question. If heterogeneity of early prophylactic treatment were added to the multi‐variable predictive model, then the model would have been of much greater clinical value and been able to provide true individual scar prevention.

To conclude, Huang et al. have contributed very well by assessing the etiology of the injury and identifying it as a predictor of injury outcome at an early stage and have used it as a risk stratification tool for developing a clinically applicable model. Acknowledging, however, that other factors, such as nutritional status, systemic factors of wound healing, and treatment variation were not considered, it is proposed that studies in the future should consider these in addition. Incorporating these factors along with injury etiology in a comprehensive predictive model can minimize potential bias and improve its accuracy of prediction of scar outcomes. In addition, it will improve the model's clinical utility, generalizability, and evidence‐based scar management improved for each individual. The authors deserve praise for their valuable contributions and we look forward to future studies in this area.

## Author Contributions

Meerab Akhtar Abbasi and Kumar Singh contributed equally to this work, including literature review, drafting of the manuscript, and critical revisions. Maheen Hamid supervised the study and critically reviewed the manuscript for important intellectual content. All authors read and approved the final version of the manuscript.

## Funding

The authors have nothing to report.

## Ethics Statement

The authors have nothing to report.

## Conflicts of Interest

The authors declare no conflicts of interest.

## Data Availability

Data sharing not applicable to this article as no datasets were generated or analysed during the current study.
